# The impact of audit and feedback on nodal harvest in colorectal cancer

**DOI:** 10.1186/1471-2407-11-2

**Published:** 2011-01-03

**Authors:** Geoffrey A Porter, Robin Urquhart, Jingyu Bu, Paul Johnson, Eva Grunfeld

**Affiliations:** 1 Department of Surgery, Dalhousie University and QEII Health Sciences Centre, Halifax, Nova Scotia, Canada; 2 Cancer Outcomes Research Program, Cancer Care Nova Scotia, Halifax, Nova Scotia, Canada; 3 Surveillance and Epidemiology Unit, Cancer Care Nova Scotia, Halifax, Nova Scotia, Canada; 4 Ontario Institute for Cancer Research and Cancer Care Ontario, Toronto, Ontario, Canada; 5 Department of Family and Community Medicine, University of Toronto, Toronto, Ontario, Canada

## Abstract

**Background:**

Adequate nodal harvest (≥ 12 lymph nodes) in colorectal cancer has been shown to optimize staging and proposed as a quality indicator of colorectal cancer care. An audit within a single health district in Nova Scotia, Canada presented and published in 2002, revealed that adequate nodal harvest occurred in only 22% of patients. The goal of this current study was to identify factors associated with adequate nodal harvest, and specifically to examine the impact of the audit and feedback strategy on nodal harvest.

**Methods:**

This population-based study included all patients undergoing resection for primary colorectal cancer in Nova Scotia, Canada, from 01 January 2001 to 31 December 2005. Linkage of the provincial cancer registry with other databases (hospital discharge, physician claims data, and national census data) provided clinicodemographic, diagnostic, and treatment-event data. Factors associated with adequate nodal harvest were examined using multivariate logistic regression. The specific interaction between year and health district was examined to identify any potential effect of dissemination of the previously-performed audit.

**Results:**

Among the 2,322 patients, the median nodal harvest was 8; overall, 719 (31%) had an adequate nodal harvest. On multivariate analysis, audited health district (p < 0.0001), year (p < 0.0001), younger age (p < 0.0001), non-emergent surgery (p < 0.0001), more advanced stage (p = 0.008), and previous cancer history (p = 0.03) were associated with an increased likelihood of an adequate nodal harvest. Interaction between year and audited health district was identified (p = 0.006) such that the increase in adequate nodal harvest over time was significantly greater in the audited health district.

**Conclusions:**

Improvements in colorectal cancer nodal harvest did occur over time. A published audit demonstrating suboptimal nodal harvest appeared to be an effective knowledge translation tool, though more so for the audited health district, suggesting a potentially beneficial effect of audit and feedback strategies.

## Background

In Canada, it is estimated there will be 22,500 new cases of colorectal cancer in 2010. More than 9,100 will die of the disease, making it the second most common cancer-causing death [1]. Survival is clearly related to stage of disease at diagnosis; the status of lymph nodes is a critical discriminator of stage, particularly in discriminating patients with stage II and stage III disease [2]. The use of adjuvant therapies has been clearly shown to improve survival for stage III patients, but less conclusively for stage II patients. Thus, nodal status often plays an important role in determining the need for, and the potential benefit from, such adjuvant therapy [3-6].

Many studies over the past 15 years have demonstrated the importance of an adequate nodal harvest in colorectal cancer, and specifically that node positivity rates increase with increased nodal harvest [7-12]. Moreover, improved survival among patients with greater nodal harvests has also been reproducibly demonstrated [13-18]. Although there exists some variability in the number of lymph nodes suggested for accurate staging [19], several organizations have advocated that a minimum of 12 lymph nodes are required. A minimum of 12 lymph nodes has been approved by the National Quality Forum as a quality indicator for colorectal cancer care, and has subsequently become a focus for quality and pay for performance initiatives [20].

Like others, we demonstrated suboptimal nodal harvest in colorectal cancer specimens approximately a decade ago [12]. This work, published in 2002, demonstrated that nodal harvest was adequate (≥ 12 lymph nodes) in only 22% of patients in a single health district. The findings were presented in multiple forums within the specific institution's health district as part of an audit and feedback strategy, over 2001-2003.

The goal of our current study was to identify factors associated with adequate nodal harvest in the entire province of Nova Scotia, and to specifically examine the impact of our prior audit and feedback strategy on subsequent nodal harvest.

## Methods

This population-based study included all patients over the age of 20 diagnosed with colorectal cancer in the province of Nova Scotia from January 1^st ^2001 to December 31st 2005, and who underwent resection. The assembly of this cohort was performed based on a linkage of the provincial cancer registry with other administrative databases including hospital discharge data, physician claims data, and national census data, which is described in detail elsewhere [21]. This linked dataset provided clinicodemographic, diagnostic and treatment data on all patients with a diagnosis of colorectal cancer in the province.

From this dataset, we included all patients undergoing surgical resection based on procedure codes (Table 1). We excluded stage IV patients (N = 394), patients in whom no lymph nodes were identified in the specimen (N = 75), and patients with rectal cancer receiving preoperative long-course radiotherapy with or without chemotherapy (N = 80) due to its known negative effect on nodal harvest [22]. The remaining study cohort was comprised of 2,322 patients. Collaborative staging was applied to all patients within the cohort [23] and was the source of nodal harvest information.

**Table 1:** Surgical procedure codes.

Code	Description
**Canadian Classification of Health Interventions (CCI) codes (2001-onward)**	
1.NM.87.^^ (except 1.NM.87.BA, 1.NM.87.DA, 1.NM.87.LA)	Excision partial, large intestine.
1.NM.89.^^	Excision total, large intestine
1.NM.91.^^	Excision radical, large intestine.
1.NQ.87.^^ (except 1.NQ.87.BA-FA and 1.NQ.87.BA, 1.NQ.87.DA, 1.NQ.87.CA, 1.NQ.87.PB, 1.NQ.87.PF)	Excision partial, rectum
1.NQ.89.^^	Excision total, rectum
**Physicians Billings procedure codes**	
MASG-57.52	Partial excision of large intestine:Terminal ileum, cecum an descending colon
MASG-57.53	Right hemicolectomy
MASG-57.55	Left hemicolectomy
MASG-57.59	Other partial excision of large intestine
MASG-57.6	Total colectomy
MASG-60.52	Other anterior resection (rectum)
MISG-60.52A	Other anterior resection (rectum)-lower anterior resection
MASG-60.55	Hartmann resection (rectum)

In 2002, we published a retrospective chart review study examining nodal harvest from colorectal cancer resections within a single Nova Scotia health district over a four-year period (1998-2001). This work was disseminated via presentations in forums in the health district where the study was performed, which was also the only academic health center in the province. These forums included Pathology Grand Rounds, Multidisciplinary Gastroenterology Rounds, Cancer Centre Clinical Site Team Tumor Board meeting, and Department of Surgery Research Day. All such forums were within the health district where the study was performed. All surgeons and pathologists involved in colorectal cancer care in the health district were exposed to at least one presentation. These presentations were focused on the results of the study, and did not advocate for any specific advance pathologic technique of increasing lymph node harvest such as fat-clearing xylose. No other provincial dissemination activities were undertaken. The study was also presented as an oral presentation at the Society of Surgery for the Alimentary Tract in June 2002 and was published in the Journal of Gastrointestinal Surgery in November 2002 [12]. No other specific intervention was undertaken to improve nodal harvest within the province of Nova Scotia over the time period of the current study (2001-2005).

For the current study, we defined an adequate nodal harvest as at least 12 lymph nodes identified within the specimen. Health care in the province of Nova Scotia is delivered within nine health districts, all of which have at least one institution providing surgical treatment for colorectal cancer. The rate of adequate nodal harvest according to health district was examined over time. We used multivariate logistic regression to identify factors independently associated with nodal harvest. *A priori *we planned to examine the interaction between health district and year on adequate nodal harvest rates in the province; this analysis was designed to identify any significant *disproportionate *improvement in nodal harvest over time in a specific health district. Statistical significance was set at p < 0.05 and all analyses were performed using SAS (Durham, North Carolina).

This study received full approval by Capital District Health Authority Research Ethic Board (#CDHA-RS/2008-049), and all required procedures related to patient confidentiality and privacy were maintained. Given the retrospective administrative database methodology, no patient-level informed consent was required.

## Results

Descriptive characteristics of the study cohort (N = 2,322) are found in Table 2. Of the study patients, 35.2%s resided in the health district in which the audit and feedback was provided, while 64.8% resided in one of the other eight health districts. Overall, the median nodal harvest amongst all patients was eight (mean 9.9; interquartile range 4-13). Node positivity rates increased with nodal harvest, as shown in Table 3 (p < 0.0001).

**Table 2:** Distribution of clinicodemographic characteristics of the study cohort (N = 2322).

	*N*	%
Age (yrs)		
20-49	128	5.5
50-64	600	25.8
65-74	646	27.8
≥ 75	948	40.8
Sex		
Female	1118	48.1
Male	1204	51.9
Tumor location		
Right colon	1010	43.5
Left colon	628	27.0
Rectum	657	28.3
Unknown/NOS	27	1.2
Cancer history		
No	1964	84.6
Yes	358	15.4
Surgery		
Emergent	436	18.8
Non-emergent	1886	81.2
Stage		
I	478	20.6
II	963	41.5
III	846	36.4
Unknown	35	1.5
Grade		
Well differentiated	206	8.9
Moderate	1546	66.6
Poorly differentiated	339	14.6
Unknown/NOS	231	9.9
DHA		
1 (no audit and feedback)	185	8.0
2 (no audit and feedback)	186	8.0
3 (no audit and feedback)	213	9.2
4 (no audit and feedback)	180	7.8
5 (no audit and feedback)	107	4.6
6 (no audit and feedback)	125	5.4
7 (no audit and feedback)	126	5.4
8 (no audit and feedback)	382	16.5
9 (audit and feedback)	818	35.2

**Total**	2322	100

**Table 3:** Association between lymph node harvest (by quartiles) and incidence of nodal metastases.

Lymph node harvest	N	% with ≥ 1 positive node(s)
1-3	470	16.0%
4-7	576	35.9%*
8-12	584	43.5%*
>12	620	42.9%*

* p<0.0001 compared to 1-3 nodes

**Figure 1:**
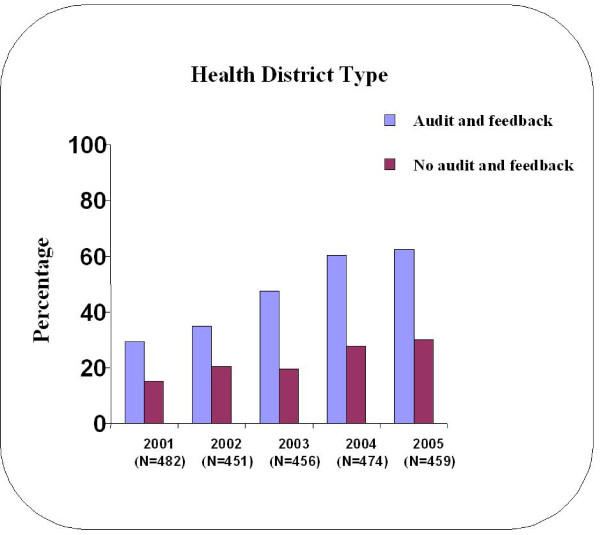
**Percentage adequate nodal harvest by year, according to Health District type (N = 2322)**.

For the entire study cohort, there was an increase in number of nodes harvested by year (Table 4). According to the *a priori *threshold of ≥ 12 lymph nodes, 719 (31%) of the 2,322 patients had an adequate nodal harvest. Figure 1 depicts the rate of adequate nodal harvest rates by year, stratified according to audit and feedback vs. non-audit and feedback health district. There was an increase in the rate of adequate nodal harvest on univariate analysis over time among both audit and feedback (p < 0.0001) and non-audit and feedback (p < 0.0001) health district. This rate increased from 15.3% in 2001 to 30.0% in 2005 in the non-audit and feedback health district, whereas within the audited health district, the adequate nodal harvest rate rose from 29.4% to 62.3% for the same time period.

**Table 4:** Number of lymph nodes by year for entire study cohort (N = 2322).

Year	Median	Interquartile range	Mean
2001	6	3-11	8
2002	8	4-12	8.9
2003	8	5-14	9.9
2004	9	5-15	11.0
2005	9	5-16	11.5

**All**	8	4-13	9.9

Table 5 summarizes the univariate and multivariate analyses of factors associated with an adequate nodal harvest. Younger age, non-emergent surgery, more advanced stage, absence of a previous cancer history, and more recent year were all associated with an increased likelihood of adequate nodal harvest. Controlling for these factors, patients who underwent surgery within the audit and feedback health district were 3.23 times more likely to have an adequate nodal harvest than patients who underwent resection in one of the non-audit and feedback health district. Of note, we were unable to assess hospital volume or type (academic vs. community) in this multivariate analysis due to significant collinearity with the audit and feedback health district variable (variance inflation factor > 10) [24].

**Table 5:** Univariate and multivariate analysis of factors associated with an adequate (≥ 12) nodal harvest (N = 2322).

		*Univariate*			*Multivariate*	
	OR	95% CI	p	OR	95% CI	p
Age			0.0006			< 0.0001
20-49	1			1		
50-64	0.78	0.53-1.15		0.82	0.53-1.26	
65-74	0.70	0.46-1.00		0.70	0.45-1.07	
≥ 75	0.54	0.37-0.78		0.49	0.32-0.75	
Sex			0.03			0.11
Female	1			1		
Male	0.83	0.69-0.98		0.85	0.70-1.04	
Tumor location			< 0.0001			< 0.0001
Right colon	1			1		
Left colon	0.58	0.47-0.72		0.54	0.42-0.69	
Rectum	0.61	0.49-0.76		0.52	0.40-0.66	
Unknown/NOS	0.59	0.59-1.41		0.79	0.31-1.97	
Cancer history			0.02			0.03
No	1			1		
Yes	0.74	0.57-0.95		0.74	0.56-0.98	
Year (continuous variable)	1.29	1.20-1.36	< 0.0001	1.36	1.27-1.45	< 0.0001
Surgery			0.02			< 0.0001
Elective	1			1		
Emergent	0.75	0.59-0.95		0.60	0.46-0.77	
Stage			< 0.0001			0.008
I	1			1		
II	1.89	1.45-2.45		1.93	1.50-2.59	
III	2.31	1.78-3.01		2.45	1.83-3.28	
Unknown	0.24	0.06-1.02		0.30	0.07-1.33	
Health District			< 0.0001			< 0.0001
No audit and feedback	1			1		
Audit and feedback	2.88	2.40-3.46		3.23	2.65-3.94	

When we examined the impact of health district over time on the adequacy of nodal harvest, there was a significant interaction. The improvement in nodal harvest over time was significantly *greater *(p = 0.007) within the audit and feedback health district than the improvement in the non-audit and feedback health district (i.e. although a significant improvement was identified in *all *health districts, this improvement was significantly greater in the audit a feedback health district).

## Discussion

Although several studies have shown improvement of colorectal cancer nodal harvest, there exists no consensus on mechanism(s) responsible for such improvement [25,26]. To our knowledge, this study is the first to specifically suggest a positive association between audit and feedback on adequacy of nodal harvest in colorectal cancer.

The use of audit and feedback to improve clinical practice has been studied across a range of health care settings, with systematic reviews demonstrating only modest improvements in practice [27,28]. The effectiveness of audit and feedback may be greater, however, when baseline adherence to a recommended practice is low [28]. In colorectal cancer, prior research has demonstrated suboptimal nodal harvest [12,29-31], with Wright and colleagues [32] demonstrating knowledge 'gaps' between current and best practices. However, traditional continuing medical education (CME) has been shown inadequate at bridging the gap between current practice and best available evidence [33].

In Ontario, Canada, a multi-faceted CME strategy, which included didactic teaching, a gastrointestinal tumour site retreat, informal opinion leadership, performance feedback, and development of a synoptic pathology report, was associated with improved colon cancer staging and nodal harvest at one tertiary cancer centre [34]. Although this overall strategy was effective, its multimodal nature makes it difficult to determine which change techniques were of most benefit. A subsequent Ontario-wide randomized controlled trial demonstrated that a standardized lecture given by formal opinion leaders improved nodal harvest in colon cancer, but academic detailing and provision of a toolkit yielded no further improvements [26].

The majority of the factors we identified to be associated with nodal harvest were consistent with previous literature. Specifically, an adequate nodal harvest is more common in right colon lesions, non-emergent surgery, and higher stage lesions [30,35,36]. We identified a modest association between previous cancer history and lower rate of adequate nodal harvest, a finding not reported previously. Although we have no definitive explanation for this finding, we hypothesize that, at least in part, it may be related to previous abdominal surgery in these patients and subsequent associated challenges in extensive mesenteric resection.

Previous studies have suggested that hospital characteristics such as volume, academic status, or presence of multidiciplinary teams may impact on quality of care in colorectal cancer. We are limited in our ability to fully evaluate these factors in our study given that the audit and feedback health district was also the highest volume centre, the only academic center, and coordinated multidisciplinary case conferences in the province.

A major strength of our study is the assessment of lymph node harvest and associated changes over time for a 5-year population-based, fully staged cohort of colorectal cancer patients. However, there are some limitations. Firstly, the methodology of this work involves the use of linked administrative data, thus limiting the ability to identify the actual clinical factors contributing to nodal harvest. The extent and technique of surgical resection and the specific nodal identification technique used by a pathologist are examples of such potential contributory factors that we are unable to examine with our data sources [37-39]. Specifically, although the audit and feedback strategy did not advocate for any specific advanced techniques of pathologic assessment (e.g. fat-clearing xylose), we are unable to determine if this occurred. Secondly, although we have clearly demonstrated an increase in nodal harvest in the audit and feedback health district, more marked over time, true causation cannot be proven in such a study design. Our data limits us in truly delineating the technical changes made (including whether the improvements were based on surgical or pathological technique, or both) that improved nodal harvest disproportionately in the audit and feedback health district. Finally, the acceptance of nodal harvest as a quality indicator of CRC care implies an association with patient outcome. Neither survival nor recurrence was not evaluated in this study for data availability reasons, but clearly is of utmost importance and is required in future studies.

## Conclusions

In conclusion, this study demonstrated that there were clear improvements in CRC nodal harvest that occurred over the time period of the study. Dissemination of a published audit demonstrating suboptimal nodal harvest appeared to be an effective tool. This suggests a potential beneficial effect of audit and feedback strategies to improve colorectal cancer care.

## Competing interests

The authors declare that they have no competing interests.

## Authors' contributions

GP, RU. PJ. EG participated in the design of the study. RU, JB assisted with the acquisition of data. GP, RU, JB, PJ, EG participated in the analysis and interpretation of data. GP, JB, RU helped to draft the manuscript. GP, RU, JB, PJ, EG participated in the critical revision of the manuscript. All authors read and approved the final manuscript.

## Pre-publication history

The pre-publication history for this paper can be accessed here:

http://www.biomedcentral.com/1471-2407/11/2/prepub
